# Comprehensiveness of online sources for patient education on otosclerosis

**DOI:** 10.3389/fsurg.2024.1327793

**Published:** 2024-01-24

**Authors:** Ahmet Adnan Cırık, Yeşim Esen Yiğit, Ahmet Mahmut Tekin, Yaşar Kemal Duymaz, Şamil Şahin, Burak Erkmen, Vedat Topsakal

**Affiliations:** ^1^ Department of Otolaryngology, Umraniye Training and Research Hospital, University of Health Sciences, Istanbul, Türkiye; ^2^ Department of Otolaryngology and Head & Neck Surgery, Vrije Universiteit Brussel, University Hospital UZ Brussel, Brussels Health Campus, Brussels, Belgium; ^3^ Private Practitioner, Istanbul, Türkiye; ^4^ Sancaktepe Martyr Prof Dr Ilhan Varank Training and Research Hospital Department of Otolaryngology, University of Health Sciences, İstanbul, Türkiye

**Keywords:** otosclerosis, readability, comprehensiveness, online sources, patient education

## Abstract

**Purpose:**

This study aimed to assess the readability indices of websites including educational materials on otosclerosis.

**Methods:**

We performed a Google search on 19 April 2023 using the term “otosclerosis.” The first 50 hits were collected and analyzed. The websites were categorized into two groups: websites for health professionals and general websites for patients. Readability indices were calculated using the website https://www.webfx.com/tools/read-able/.

**Results:**

A total of 33 websites were eligible and analyzed (20 health professional-oriented and 13 patient-oriented websites). When patient-oriented websites and health professional-oriented websites were individually analyzed, mean Flesch Reading Ease scores were found to be 52.16 ± 14.34 and 46.62 ± 10.07, respectively. There was no significant difference between the two groups upon statistical analysis.

**Conclusion:**

Current patient educational material available online related to otosclerosis is written beyond the recommended sixth-grade reading level. The quality of good websites is worthless to the patients if they cannot comprehend the text.

## Introduction

In an era of rapidly developing technological innovations, patients should have easy access to information about their disease on the internet. One in five people use the internet to research detailed medical information about their disease even before consulting an otolaryngologist (1). Moreover, even health professionals use the internet to gather information about a specific disease. These information sources must therefore provide reliable, easily readable, and understandable texts (2).

Otosclerosis, characterized by an abnormally high pace of bone remodeling in the otic capsule, is a cause of progressive hearing loss. Its incidence is 0.1% in the Caucasian population. It is much less frequent in Asians and Native Americans. Patients with otosclerosis generally present with tinnitus and progressive conductive hearing loss (3). The progression of sensorineural hearing loss is independent of age and sex (4). Ten percent of patients also experience sensorineural hearing loss due to cochlear involvement (5). It is diagnosed with clinical presentation, pure-tone audiogram results, stapedial reflexes, and often high-resolution CT scans (3).

A strong genetic origin is suspected, and generally, it is accepted as an autosomal dominant disease with variable penetrance. For a long time, no animal models were available, and it is exclusively a human disease. Eight different loci have been discovered so far in eight distinct families (6). The Foxl 1 gene has been identified as a monogenic cause, and about 10 families have been localized with regions of interest for further study. It is anticipated that additional disease-causing genes will be published soon (7, 8). ACAN is another candidate gene associated with the etiology of otosclerosis (6). While the SERPINF1 gene was found to lack sufficient evidence in relation to the etiology of otosclerosis (9), a recent publication has suggested that a variant of SMARCA4 might be the cause of otosclerosis (10).

Oral sodium fluoride therapy for several months was thought to slow down the bone turnover rate. However, bony structural alterations have no known cure yet. In addition, there have been claims that fluoride in tap water should be enough for an equal therapeutic effect (11). The rehabilitation of conductive hearing loss can involve hearing aids or surgical treatment (12). Patients may start with hearing aids, and if they are not sufficient, stapedotomy is a well-standardized bypass option for addressing conductive hearing loss (13). Hearing aids are commonly used by many patients and carry no potential risk in their use, but they can often pose a burden to users. However, patients are mostly not well informed or even incapable of choosing surgery or hearing aids. A trial period with hearing aids is already a tool to help them decide, and well-standardized readable information on the internet could serve as guidance.

Furthermore, otosclerosis surgery comes with its own set of surgical risks that patients need to know in advance. Complications such as facial paralysis, perceptive hearing loss, cerebrospinal fluid leakage, prosthesis dislocation, and vertigo have been described, and most patients want to be aware of these risks (12). Considering that otosclerosis is a progressive disease, even after successful surgery (often around 40–55 years), most patients progress with sensorineural hearing loss and would need hearing aids in their later years, around the age of 70 or 80 years. So, there are many aspects for an otologist to correctly counsel every case, and vouchers, leaflets, and websites can provide significant benefits in informing patients about this disease.

Patients diagnosed with otosclerosis are very likely to search for their condition on the internet because the choice for having surgery is elective. Helping patients make correct treatment choices is a cornerstone of treatment strategies and will also help in dealing with eventual postoperative complications.

Many web sources provide information, but little is known about their quality. Assessing whether web sources use too much medical terminology and whether the layperson can understand the context is crucial. For this reason, we aim to assess the readability indices of websites including educational materials on otosclerosis.

## Materials and methods

We performed a Google search on 19 April 2023 using the term “otosclerosis.” The first 50 hits were collected and analyzed. After 50 clicks, the quality of websites severely deteriorated, and many other studies on readability indices stopped beyond the first 50 hits (2, 14). Websites containing only videos, those in a non-English language, and those with duplicate results were among the exclusion criteria; in addition, academic articles were excluded. The websites were categorized into two groups: websites for health professionals and general websites for patients. The categorization was done by considering the way readers were addressed. If words such as “your symptoms, your complaints” were used, these were categorized as general websites for patients. However, if medical jargon was used or words such as “your patient” were used, then these were categorized as websites for healthcare professionals. The website content was copied to Microsoft Word. Links, resources, videos, images, and advertisements were removed.

Readability indices were calculated using the website https://www.webfx.com/tools/read- able/. Readability indices have different subtypes: Flesch Reading Ease (FRE), Flesch–Kincaid grade level (FKGL), Gunning–Fog index (GFI), simple measure of Gobbledygook (SMOG), Coleman–Liau index (CLI), and automated readability index (ARI). Each score has a different calculation. FRE and FKGL use sentence and word length in the calculation. FKGL is the most considered score. GFI is used to show clarity and simplicity (complex words are used in the calculation). SMOG uses polysyllabic count in the calculation, and it indicates complete comprehension. ARI and CLI use characters in the calculation rather than syllables like most of the other formulas. The automatically calculated results were collected. When using the website, it was noted that headings/subheadings ended with a period, preventing them from merging with the upcoming sentence. In addition, sentences with bullet points ended with a period (if there was no period at the end of the sentence, we added it); therefore, these sentences did not merge with the upcoming sentence.

Permission from an ethical committee was not required since the research included only publicly accessible data.

### Statistical method

For descriptive statistics, mean, standard deviation, median, minimum, maximum value frequency, and percentage were calculated. The Kruskal–Wallis test was used for the comparison of quantitative data. SPSS 28.0 was preferred for statistical analyses.

## Results

A total of 33 websites were eligible and were copied into Microsoft Word. Of these websites, 13 were patient-oriented websites and 20 were health professional-oriented. The scores of all websites and the countries of their origin are listed in Table 1.

**Table 1:** Scores of websites and their country of origin.

	Website	FRE	FKGL	GFS	SMOG	CLI	ARI	Country
	General websites for patients							
1	https://www.webmd.com/cold-and-flu/ear-infection/otosclerosis-facts	61.9	8.2	9.4	7.9	11	7.2	USA
2	https://www.betterhealth.vic.gov.au/health/conditionsandtreatments/ears-otosclerosis	37.2	14.1	16.8	12.2	13.7	14.4	Australia
3	https://www.healthdirect.gov.au/otosclerosis	60.3	8.3	11	8.2	11.3	7.3	Australia
4	https://patient.info/ears-nose-throat-mouth/hearing-problems/otosclerosis	58.9	8.6	11.1	8.3	11.9	7.8	UK
5	https://medlineplus.gov/ency/article/001036.htm	58.1	9	10.9	8.8	11.9	8.5	USA
6	https://www.hear-it.org/otosclerosis	47.3	10.6	13.1	9.9	14	10.4	USA
7	https://vestibular.org/article/diagnosis-treatment/types-of-vestibular-disorders/otosclerosis/	15.4	15.8	18.7	13.5	19.1	15.9	USA
8	https://www.bmc.org/patient-care/conditions-we-treat/db/otosclerosis	49.6	10.2	12.8	9.7	13.2	9.6	USA
9	https://rnid.org.uk/information-and-support/ear-health/common-ear-problems/otosclerosis/	64.4	8.5	10.2	7.7	10.8	8.4	UK
10	https://www.healthyhearing.com/report/53072-Otosclerosis	52.6	9	11	8.8	13.3	8	USA
11	https://www.healthline.com/health/otosclerosis#summary	44.9	11.2	13.9	10.2	14.2	11	USA
12	https://www.drugs.com/cg/otosclerosis.html	72.4	6.4	9	6.7	10.8	6.4	New Zealand
13	https://www.ndcs.org.uk/information-and-support/childhood-deafness/causes-of-deafness/otosclerosis/	55.1	9.1	12	8.9	12.7	8.5	UK
	Websites for health professionals							
1	https://www.nhs.uk/conditions/otosclerosis	27.3	19.9	22.4	14.6	11.6	21.6	UK
2	https://www.nidcd.nih.gov/health/otosclerosis	44	11.6	14.1	10.5	14.6	12	USA
3	https://my.clevelandclinic.org/health/diseases/22033-otosclerosis	51.2	9.2	11.7	8.8	14.2	8.7	USA
4	https://www.pennmedicine.org/for-patients-and-visitors/patient-information/conditions-treated-a-to-z/otosclerosis	57.7	9.1	11	8.8	12	8.6	USA
5	https://www.enthealth.org/conditions/otosclerosis/	54.7	9.2	11.9	9	12.8	8.7	USA
6	https://med.stanford.edu/ohns/OHNS-healthcare/earinstitute/conditions-and-services/conditions/otosclerosis.html	52.3	9.9	11.6	9.1	12.7	9.3	USA
7	https://www.mountsinai.org/locations/center-hearing-balance/conditions/otosclerosis	49.3	10.4	13.7	10.1	13.1	9.7	USA
8	https://www.teomandal.com/en/otosclerosis-calcification-of-the-middle-ear	40.3	13.6	16.1	11.9	14.1	14.4	Türkiye
9	https://www.american-hearing.org/disease/otosclerosis/	36	12.7	14.9	11.1	15.7	12.7	USA
10	https://ufhealth.org/otosclerosis	59	8.9	10.9	8.6	11.8	8.4	USA
11	https://www.ear.co.nz/otosclerosis	48.9	10.3	12.7	9.7	13	9.3	New Zealand
12	https://entlv.com/ent/ear/otosclerosis/	48.3	10.7	13.2	10.1	13.4	10.3	USA
13	https://hamiddjalilianmd.com/conditions/otosclerosis/	55.2	9.7	11.8	8.9	12.4	9.5	USA
14	https://www.masseyeandear.org/conditions/otosclerosis	38.1	12.6	12.6	10.5	16.1	13.5	USA
15	https://www.tgh.org/institutes-and-services/conditions/otosclerosis	21.7	18.1	19	14.5	16.8	20.5	USA
16	https://www.pediatricentillinois.com/pediatric/what-you-should-know-about-otosclerosis/	46.5	11	13	10	13.3	10.3	USA
17	https://www.earassociates.com/conditions-otosclerosis-san-jose-ca.html	40.3	11.7	14.1	10.7	14.4	10.9	USA
18	https://www.specsavers.co.uk/ear-health/otosclerosis	52.7	11.5	13.4	9.6	12.1	12	UK
19	https://www.entuk.org/patients/conditions/14/otosclerosis_and_stapedotomy_update/	57.4	8.7	11.5	8.6	12.1	7.8	UK
20	https://www.tampabayhearing.com/ear-education/auditory-education/otosclerosis/	51.5	9.9	12.1	9.3	13.2	9.5	USA

FRE, Flesh Kincaid reading ease; FKGL, Flesh–Kincaid grade level; GFS, gunning fog score; SMOG, simple measure of the Gobbledygook; CLI, Coleman–Liau index; ARI, automated readability index.

The mean FRE score of all 33 websites was 48,803 ± 12,037 (10th–12th grades). Mean FKGL was 10.839 ± 2.852 (10th–12th grades). The mean Gunning–Fog index was 13.08 ± 2.851 (13th–14th grades). The mean SMOG index was 9.854 ± 1.813 (9th–10th grades). Mean CLI was 13.251 ± 1.790 (13th–14th grades). Mean ARI was 10.639 ± 3.504 (10th–12th grades) (Table 2).

**Table 2:** Descriptive statistics.

		*N*	Minimum	Maximum	Mean	SD
General websites for patients	Flesh Kincaid reading ease	13	15.40	72.40	52.1615	14.34146
	Flesh Kincaid grade level	13	6.40	15.80	9.9231	2.55478
	Gunning–Fog score	13	9.00	18.70	12.3000	2.81721
	Smog index	13	6.70	13.50	9.2923	1.86031
	Coleman–Liau index	13	10.80	19.10	12.9154	2.22218
	Automated readability index	13	6.40	15.90	9.4923	2.83033
	Total number	13				
Websites for health professionals	Flesh Kincaid reading ease	20	21.70	59.00	46.6200	10.07021
	Flesh Kincaid grade level	20	8.70	19.90	11.4350	2.93783
	Gunning–Fog score	20	10.90	22.40	13.5850	2.82718
	Smog index	20	8.60	14.60	10.2200	1.72950
	Coleman–Liau index	20	11.60	16.80	13.4700	1.46722
	Automated readability index	20	7.80	21.60	11.3850	3.76022
	Total number	20				
Results of all two groups	Flesh Kincaid reading ease	33	15.40	72.40	48.8030	12.03751
	Flesh Kincaid grade level	33	6.40	19.90	10.8394	2.85219
	Gunning–Fog score	33	9.00	22.40	13.0788	2.85107
	Smog index	33	6.70	14.60	9.8545	1.81264
	Coleman–Liau index	33	10.80	19.10	13.2515	1.79045
	Automated readability index	33	6.40	21.60	10.6394	3.50446
	Total number	33				

When patient-oriented and health professional-oriented websites were individually analyzed, mean FRE scores were found to be 52.16 ± 14.34 and 46.62 ± 10.07, respectively. Mean FKGL scores were 9.923 ± 2.554 and 11.435 ± 2.938, respectively. Mean Gunning–Fog indices were 12.3 ± 2.817 and 13.585 ± 2.837, respectively. Mean Smog indices were 9.292 ± 1.86 and 10.220 ± 1.729, respectively. Mean CLI scores were 12.915 ± 2.222 and 13.47 ± 1.467, respectively. Mean ARI scores were 9.492 ± 2.820 and 11.385 ± 3.760, respectively. There was no significant difference between the two groups upon statistical analysis (Table 3).

**Table 3:** Test statistics.

	Flesh–Kincaid reading ease	Flesh–Kincaid grade level	Gunning–Fog score	Smog index	Coleman–Liau index	Automated readability index
Kruskal–Wallis *H*	3,046	4,561	2,984	3,595	1,890	3,952
df	2	2	2	2	2	2
Asymp. Sig. (*p*-value)	218	102	225	166	389	139

## Discussion

In our study, our aim was to assess the readability indices of websites including educational materials on otosclerosis. Despite the proven effectiveness and safety of hearing aids, patients often look for alternative therapies online, especially if they are less visible. Stapedectomy is still known for resulting in sensorineural hearing loss after surgery in 1 in 100 cases, a concern frequently noted by patients (15). While no surgery is risk-free, patients look for data on the internet to help them decide on their customized therapy.

Today, because of technology, patients have easy access to information about their health issues online. Most of the patients use the internet to search for medical information regarding the specifics of their condition before meeting with an otolaryngologist. As a result, the internet significantly influences patients’ treatment decisions. In addition, healthcare professionals may also resort to online platforms to seek information about diseases that may be unfamiliar or novel to them. Strikingly, anyone can write anything on the internet, and there is not much regulation or criteria for either professional-oriented or patient-oriented disease informative websites. A primary requirement to reach a general readers audience to which patients may belong would be to look for the readability of the texts of these sources.

The American Medical Association (AMA) and the National Institutes of Health (NIH) propose that patient information be prepared at the eighth- and sixth-grade levels, respectively (16, 17). A small fraction of medical resources is written at a grade level suitable for the public, as evidenced by the readability of patient educational materials from high-impact medical publications (16). For appropriate information, patient educational materials must be understandable. However, in our study, overall FKGL was found to be 10th grade, which is above the recommended reading level. When each group was individually analyzed, patient-oriented websites were found to be of the 9th level, but health professional websites were calculated as the 11th level. Compared to the desired level 6, both are too demanding for their audience. Patient-oriented websites were easier to read, however there was not a significant statistical difference (*p* > 0.05) (Table 2). For professionals, the high-grade reading level is an expected and scrutable result because of their higher literacy rate. However, for an average person, the results are above than average readability level (sixth grade). Moreover, patients may choose to read from health professional-oriented websites, which are not only available to professionals (2). Consequently, such websites could potentially confuse patients lacking a medical background.

Many readability studies have been reported both in otolaryngology and other medical fields. Eloy et al. analyzed 262 patient educational materials from major otolaryngology association websites and used 10 different readability scales. They found that FKGL ranges from 9.7 to 17.1 (18). Therefore, all available documentation was above the suggested fourth- to sixth-grade level when using readability as an estimate for comprehension. Patel et al. evaluated 31 patient educational materials on thyroid surgery and found that the mean FKGL was 10.4 (19). None of them were found to be easy to read. In all, 16 articles were categorized as average difficulty using FKGL. Cherla et al. analyzed 31 online educational materials on endoscopic sinus surgery and highlighted that only one article’s FKGL was close to sixth grade; the mean score was 10.7 (20). Kong and Hu searched 50 online tracheostomy sources and found that the mean FKGL was 8.3 (2). They categorized the websites into two subgroups: professional-oriented and patient-oriented. FKGL scores of professional-oriented websites were found to be higher than patient-oriented websites. Furthermore, in our previous study, hereditary hearing loss was researched on this aspect and all analyzed websites required higher reading levels than the sixth grade (21). In other fields of medicine, similar results are reported regarding readability (22–24). All these studies are highly significant because internet-based patient educational tools have allowed people to easily explore their ailments thanks to the abundance of freely available internet medical information. However, low health literacy might cause readers to misread the online material.

Overall, the readability of otosclerosis educational materials needs to improve to reach a wider audience. This could be achieved by implementing layperson's language and avoiding medical jargon. In addition, shorter sentences and simple words are preferred. The number of commonly used words may be increased. This would increase the readability scores of the educational materials. Increased readability will help patients comprehend their conditions better. Hence, patients will be more eager for their treatment and follow-up period. Patients will be more compliant with their treatment.

Our study may have some limitations, such as the fact that we only accessed Google as a search engine; however, it is the most commonly available engine for Anglo-Saxon literature for which there is a standard level of readability, determined as sixth grade.

More importantly, evaluating the readability may not be misinterpreted as good-quality information. This study did not evaluate the content of the texts nor the effect of the information on helping patients decide their treatment options. It seems logical that to reach the target, all text should be readable by a large audience from the beginning.

## Conclusion

Current patient educational material available online related to otosclerosis is written beyond the recommended sixth-grade reading level. Since the internet is progressively gaining importance in patient counseling and is becoming a tool for physicians and healthcare workers in patient and professional education regarding the diagnosis, treatment, and follow-up of diseases, readability is as important as the content of information. The quality of good websites is worthless to the patients if they cannot comprehend the text.

## Data Availability

The original contributions presented in the study are included in the article/Supplementary Material, further inquiries can be directed to the corresponding author.
